# *Pseudomonas aeruginosa* adaptation and persistence in the aspergilloma microbiome revealed by integrated multi-omics

**DOI:** 10.1093/g3journal/jkag063

**Published:** 2026-03-17

**Authors:** Matheus Mertz Ribeiro, Chan Liu, Jin-Fu Xu, Shuo Liang, Gustavo H Goldman

**Affiliations:** Faculdade de Ciências Farmacêuticas de Ribeirão Preto, Universidade de São Paulo, Ribeirão Preto, São Paulo CEP 14040-903, Brazil; Department of Respiratory and Critical Care Medicine, Shanghai Pulmonary Hospital, School of Medicine, Tongji University, Shanghai 200092, China; Institute of Respiratory Medicine, School of Medicine, Tongji University, Shanghai 200092, China; Institute of Respiratory Medicine, School of Medicine, Tongji University, Shanghai 200092, China; Department of Respiratory and Critical Care Medicine, Tongji Hospital, School of Medicine, Tongji University, Shanghai 200092, China; Department of Respiratory and Critical Care Medicine, Shanghai Pulmonary Hospital, School of Medicine, Tongji University, Shanghai 200092, China; Institute of Respiratory Medicine, School of Medicine, Tongji University, Shanghai 200092, China; Faculdade de Ciências Farmacêuticas de Ribeirão Preto, Universidade de São Paulo, Ribeirão Preto, São Paulo CEP 14040-903, Brazil; National Institute of Science and Technology in Human Pathogenic Fungi, Ribeirão Preto, São Paulo CEP 14040-903, Brazil

**Keywords:** chronic pulmonary aspergillosis, *Pseudomonas aeruginosa*, *Aspergillus fumigatus*, polymicrobial interactions, multi-omics, Fungal2026

## Abstract

Chronic pulmonary aspergillosis involves the formation of a fungal ball (aspergilloma) in lung cavities. *Pseudomonas aeruginosa* commonly co-colonizes these lesions; however, the in vivo mechanisms underlying its persistence are unknown. Using a multi-omics approach on resected aspergillomas, we defined the genomic, transcriptional, and metabolic adaptations of *P. aeruginosa* within this polymicrobial niche. We reconstructed high-quality *P. aeruginosa* genomes and identified a conserved core genome, along with accessory genes for secondary metabolism, virulence, and antimicrobial resistance. Phylogenomics revealed heterogeneous evolutionary paths among co-colonizing strains. Metatranscriptomics showed stark physiological heterogeneity, from metabolically aggressive to stress-adapted states. High expression of phenazine, quorum-sensing (PQS), siderophore, and secretion-system operons was corroborated by metabolomic detection of phenazine-1-carboxylic acid and 2-heptylquinolin-4(1H)-one, confirming active bacterial antagonism in vivo. Concurrent *Aspergillus fumigatus* transcriptomics revealed the activation of oxidative stress responses, secondary metabolism (eg fumagillin), and iron scavenging, demonstrating reciprocal competition. Host transcriptomics revealed patient-specific immune signatures that correlated with the metabolic activity of the co-colonizers. This work provides an integrated systems-level analysis of the tri-kingdom aspergilloma ecosystem. *P. aeruginosa* persistence is driven by genomic plasticity and context-dependent expression of competitive pathways, shaped within a chronic inflammatory environment. These findings redefine aspergillomas as active polymicrobial consortia, establishing a framework for targeting resilient microbial communities in chronic lung disease.

## Teaser

Research is for microbiologists and infectious disease specialists studying chronic lung infections. Ribeiro *et al*. provide an integrated view of real human infection sites, revealing how bacteria and fungi interact inside patients. Using advanced genomic and chemical profiling of surgically removed lung fungal balls, the authors found that the bacterium *Pseudomonas aeruginosa* persists alongside the fungus Aspergillus by activating specific genes for competition, like toxin production and iron scavenging. These microbes shape and respond to the patient’s unique immune environment. They conclude that these lesions are active, multispecies battlefields, not inert masses. This redefines chronic aspergillomas and highlights targets for disrupting these communities.

## Introduction

Cystic fibrosis (CF) is a life-limiting autosomal recessive disorder caused by mutations in the CFTR gene, leading to impaired epithelial ion transport and the accumulation of dehydrated, highly viscous mucus in the airways. This altered mucosal environment promotes persistent inflammation and recurrent infections that progressively damage the lung. Affecting an estimated 89,000 individuals worldwide (Ong and Ramsey 2023), CF remains dominated clinically by chronic respiratory infection, which is the primary driver of morbidity and mortality. Over time, the CF lung evolves into a heterogeneous and dynamic ecological niche shaped by chronic neutrophilic inflammation, hypoxia, and nutrient limitation, conditions that enable colonization by diverse and adaptive microbial communities (Blanchard et al. 2022). In this context, *Pseudomonas aeruginosa* and *Aspergillus fumigatus* stand out as 2 of the most consequential pathogens. Each is independently associated with accelerated lung-function decline, intensified airway inflammation, and increased mortality (Kosmidis and Denning 2015; Reynolds and Kollef 2021). Co-colonization is remarkably frequent, with up to 60% of individuals chronically infected with *P. aeruginosa* also harboring *A. fumigatus.* The simultaneous detection of these bacteria in sputum and bronchoalveolar lavage fluid underscores the prevalence and likely clinical significance of bacterial–fungal coexistence in CF (Margalit et al. 2021; de Castro et al. 2025).

The interaction between these pathogens is multifaceted, context-dependent, and deeply influenced by the host environment. *P. aeruginosa* secretes a repertoire of inhibitory molecules, phenazines such as pyocyanin, phenazine-1-carboxylic acid, and phenazine-1-carboxamide generate reactive oxygen species that damage *A. fumigatus* hyphae (Price-Whelan et al. 2006), while rhamnolipids disrupt fungal biofilms and cell wall integrity, impairing hyphal development and altering melanin deposition (Briard et al. 2017). Conversely, *A. fumigatus* counters these antagonistic pressures through high-affinity siderophore-mediated iron acquisition, ferricrocin, hydroxyferricrocin, fusarinine C, and triacetylfusarinine C, which offset *P. aeruginosa* pyoverdine-mediated iron sequestration and promote fungal competitiveness in polymicrobial environments (Sass et al. 2018). Additionally, *P. aeruginosa* quinolone signals such as PQS can suppress or enhance fungal metabolism depending on iron availability, highlighting the environmental contingency of interkingdom interactions (Williams 2007).

These microbial exchanges unfold within a host environment dominated by persistent inflammation, hyperviscous mucus, and steep oxygen gradients. Such pressures drive rapid microbial diversification and genetic adaptation. *P. aeruginosa* commonly evolves mucoid, biofilm-associated phenotypes, while *A. fumigatus* forms dense mycelial aggregates (aspergillomas) that exacerbate airway obstruction and hypoxia. The resulting polymicrobial biofilms become resilient ecosystems structured by micronutrient competition, metabolic cross-feeding, and interspecies signaling. While in vitro studies have uncovered essential mechanisms of antagonism and coexistence (Mowat et al. 2010; Shirazi et al. 2016), the in vivo genomic, metabolic, and transcriptional architecture that enables *P. aeruginosa* to persist within fungal-dominated niches remains incompletely defined.

Chronic pulmonary aspergillosis (CPA), a progressive and debilitating infectious disease, affects approximately 1.8 million individuals globally each year due to the widespread presence of Aspergillus (Reynolds and Kollef 2021; Sengupta et al. 2025). This condition typically develops in patients with underlying structural lung damage, including the residual cavities from tuberculosis or COPD (chronic obstructive pulmonary disease) (Smith and Denning 2011). A key distinction of CPA is that, unlike the invasive form of the disease, it primarily occurs in patients who are immunocompetent or only mildly immunocompromised, rather than in those with severe immunosuppression (Denning and Chakrabarti 2017; Jaggi et al. 2024). CPA is notably characterized by the development of fungal balls, or aspergillomas, within lung cavities. These formations are more than simple clusters of hyphae; they are intricate, organized biofilms. Each is a defined mass encased in a dense extracellular matrix that traps fungal filaments, immune cells, and cellular debris from the host (Kosmidis and Denning 2015). This biofilm configuration is central to the disease's severity, fueling ongoing inflammation, gradual lung tissue breakdown, and potentially fatal pulmonary hemorrhage (Evans et al. 2024). The protective matrix fosters a high degree of treatment resistance, serving as a shielded environment in which antifungal resistance can develop and relapse become common, thereby severely complicating efforts to manage the disease over the long term (Howard et al. 2009, 2013; Bongomin et al. 2020).

Previously, we investigated the multi-omic characterization of aspergillomas resected from 61 patients with CPA (Liu et al. 2026). This work defined the aspergilloma as a complex polymicrobial biofilm ecosystem, revealing key metabolic adaptations and vulnerabilities of clinical *A. fumigatus* strains within their native environment (Liu et al. 2026). It provided a new conceptual framework for developing therapies that target the pathogenic consortium and its resistant biofilm state, rather than the fungus in isolation. Here, we extended these observations by reconstructing the *P. aeruginosa* genomes of the strains that colonized the fungal balls of 3 CPA patients. By reconstructing high-quality metagenome-assembled genomes (MAGs), characterizing secondary metabolite clusters, virulence factors, and antimicrobial resistance genes, and integrating comparative pangenomics with *P. aeruginosa* reference strains, together with bacterial, fungal, and host transcriptomics and untargeted metabolomics, we delineate the metabolic, genetic, and regulatory strategies that sustain bacterial–fungal coexistence. This systems-level approach provides new mechanistic insight into chronic lung disease and offers a foundation for developing targeted therapeutic interventions against polymicrobial biofilms.

## Materials and methods

### Sample collection and processing

Fungal ball specimens were obtained intraoperatively from patients undergoing pulmonary lobectomy performed by specialized thoracic surgeons. All procedures were carried out under sterile conditions to minimize external contamination. Within 30 min of resection, fungal masses were macroscopically identified, carefully dissected from the surrounding lung tissue, and immediately aliquoted into sterile nuclease-free cryovials. Samples were flash-frozen in liquid nitrogen and stored at −80 °C until multi-omics analyses.

### Total genomic DNA extraction

Approximately 50 mg of the central and peripheral areas of the fungal ball material were used for total genomic DNA extraction following a modified CTAB protocol. Tissue was homogenized in CTAB extraction buffer and incubated with lysozyme for 2 h at 65 °C, followed by purification with phenol:chloroform:isoamyl alcohol (25:24:1) and chloroform:isoamyl alcohol (24:1). DNA was precipitated with isopropanol, washed with 75% ethanol, and resuspended in nuclease-free water. RNA contaminants were removed by RNase A treatment. DNA concentration and purity were measured with a NanoDrop One spectrophotometer, and integrity was confirmed by electrophoresis on a 1% agarose gel.

### Total RNA extraction

Total RNA was isolated from ∼50 mg of fungal ball tissue using TRIzol Reagent (Invitrogen) and mechanical disruption in a FastPrep-24 instrument. RNA precipitation efficiency was enhanced with GlycoBlue, and RNA was eluted in RNase-free water. Concentration and purity were evaluated using a NanoDrop One, and RNA integrity was assessed using an Agilent 2100 Bioanalyzer. Only samples with RIN > 7.0 were used for library construction.

### Metagenomic sequencing

DNA samples containing ≥0.2 μg were selected for library preparation. DNA was enzymatically fragmented to ∼300 bp, then end-repaired, A-tailed, adapter-ligated, and PCR-amplified. Library size distribution was assessed with a Qsep400 Bioanalyzer and quantified with Qubit 4.0. Equimolar pools were sequenced on an Illumina NovaSeq 6000 platform (paired-end 150 bp).

### Metatranscriptomic sequencing

Metatranscriptomic libraries were prepared from 10 to 100 ng of total RNA using the AccuNext Stranded Single Cell & Low Input RNA-seq Kit (AccuraBio), including depletion of both prokaryotic (16S/23S) and eukaryotic (18S/28S) rRNAs. The enriched RNA was fragmented and reverse-transcribed, with strand specificity maintained by dUTP incorporation during second-strand cDNA synthesis. Indexed libraries were amplified (12 to 15 PCR cycles), evaluated using a Bioanalyzer 2100, pooled equimolarly, and sequenced on an Illumina NovaSeq 6000 (PE150).

### Quality control and host read removal

Raw DNA and RNA reads were processed with Fastp v0.23.4 for adapter trimming, removal of low-quality bases, and filtering of reads <50 bp (Chen et al. 2018). Host-derived sequences were removed using Kraken2 v2.1.3 (Wood et al. 2019) with the SILVA rRNA database release 138 (Quast et al. 2013). High-quality filtered reads were retained for genome reconstruction and transcriptomic analysis.

### MAG reconstruction

Metagenome assembly and binning were performed using MetaWRAP v1.3.2 (Uritskiy et al. 2018). Assemblies were generated using metaSPAdes (Nurk et al. 2017), and binning was conducted with MaxBin2 (Wu et al. 2016), MetaBAT2 (Kang et al. 2019), and CONCOCT (Alneberg et al. 2014). Bins were refined within MetaWRAP, and genome quality was evaluated with CheckM (Parks et al. 2015). Only bins with ≥80% completeness and ≤5% contamination were retained. Taxonomy was assigned using SILVA rRNA release 138, and bins classified as *P. aeruginosa* were used for downstream analysis.

### Functional annotation, virulence factors, and antimicrobial resistance

Functional genome annotation was conducted using Prokka (Seemann 2014). Virulence factors were identified using the Virulence Factor Database (VFDB) and Comprehensive Antibiotic Resistance Database (CARD) via Resistance Gene Identifier (RGI) (Alcock et al. 2023). Secondary metabolite biosynthetic gene clusters (BGCs) were predicted using antiSMASH v7 (Blin et al. 2023), and additional functional annotation was performed using eggNOG-mapper v2 (Cantalapiedra et al. 2021).

### Pangenome analysis

The *P. aeruginosa* pangenome was analyzed using Roary (Page et al. 2015) from the 3 reconstructed MAGs and the reference genomes (RefSeq; Supplementary Table 2). Core genes were defined as those present in ≥95% of genomes. Visualization of gene clusters, presence–absence matrices, and pangenome distribution was performed using Anvi’o v7 (Eren et al. 2021).

### Metatranscriptomic analysis

Quality control followed the same pipeline described for DNA. Ribosomal RNA was removed using SortMeRNA v4.3.6 (Kopylova et al. 2012), and human reads were filtered by mapping against the *Homo sapiens* GRCh38.p14 genome using Bowtie2 v2.5.1 (Langmead and Salzberg 2012).

For eukaryotic transcriptomics, high-quality reads were mapped to *A. fumigatus* Af293 (GCF_000002655.1) and *H. sapiens* GRCh38 using HISAT2 v2.2.1 and quantified with StringTie2 v2.2 (Pertea et al. 2015). For bacterial transcriptomics, reads were mapped to *P. aeruginosa* PAO1 (GCF_000006765.1) using Bowtie2, and gene counts were generated with featureCounts (Liao et al. 2014). Gene expression values were normalized to TPM, and downstream functional enrichment analyses (GO, KEGG) were performed using iDEP v1.1 (Ge et al. 2018).

### Statistical analysis

All statistical analyses were performed in Python (v3.12.12) using the scipy, scikit-learn, pandas, and matplotlib libraries. Pairwise sample similarity was quantified using Spearman's rank correlation. Dimensionality reduction was conducted with principal component analysis (PCA) on log_2_-transformed TPM values. Heatmaps were generated using hierarchical clustering (Euclidean distance, Ward linkage). All data visualization (PCA, heatmaps, bar plots, and chord diagrams) was performed in Python (version 3.12.12).

## Results

### Assembly, taxonomic classification, and quality assessment of *Pseudomonas* MAGs

We previously characterized the fungal ball of 61 patients (Liu et al. 2026). Computed tomography scans and histopathology were used to visualize fungal masses and lung infections in selected patients (Liu et al. 2026). To investigate the microbiome, metabolome, and metatranscriptome of the fungal balls, excised fungal balls (54% from the left lobe and 46% from the right lobe) were collected, and some were sent for pathological staining, while others were preserved for multi-omics analysis (Liu et al. 2026). Based on 16S and ITS amplicon analysis, *A. fumigatus* dominates the fungal niche (59% of patients), bacterial co-colonization is ubiquitous, primarily by *P. aeruginosa* and *Haemophilus influenza* (Liu et al. 2026). The bacterial genus that comprises more than 90% of bacterial species is: 6 of *H. influenzae*, 5 of *P. aeruginosa*, 6 of *Neisseria*, 1 of *Nocardia*, 10 of unidentified species, and 33 of mixed species. Our main goal was to characterize fungal balls predominantly colonized by *A. fumigatus* and *P. aeruginosa*. Unfortunately, only fungal balls from 3 patients (A3, A25, and A61) provided sufficient DNA coverage to assemble a full *P. aeruginosa* genome and a sufficient number of *P. aeruginosa* and *A. fumigatus* transcripts (patients A25 and A61). Only 3 patients fulfilled these conditions and are here analyzed.

**Fig. 1.**
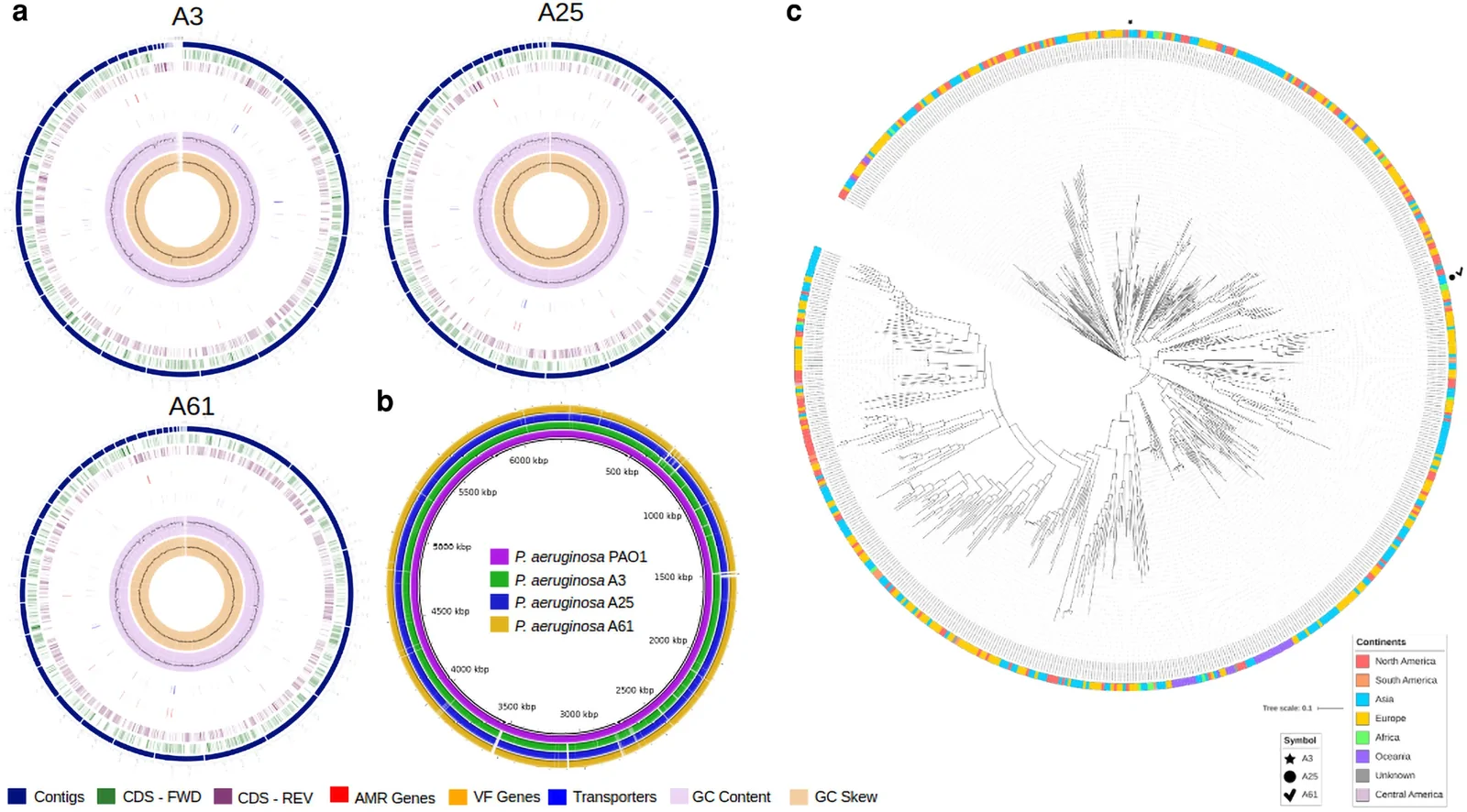
Genomic architecture and functional landscape of *Pseudomonas aeruginosa* isolates recovered from CF–aspergilloma samples. a) Whole-genome assemblies of *P. aeruginosa* strains A3, A25, and A61, showing contig organization, coding sequences (CDS) on forward and reverse strands, GC content, and GC skew, AMR genes, virulence factors (VF), and membrane transport–related genes are highlighted to illustrate genomic hotspots associated with pathogenicity and adaptation. b) BRIG comparative alignment of all sequenced isolates against the PAO1 genome. Conserved and divergent genomic regions are visualized across strains, revealing shared core architecture and isolate-specific accessory loci that may reflect niche specialization within the aspergilloma environment. c) Global pangenome analysis of 648 *P. aeruginosa* reference genomes, displaying the distribution of genomes across continents and the pangenome structure. The 3 isolates from this study are annotated with distinct symbols, allowing their placement within the global genomic diversity and highlighting their phylogeographic relationships and accessory gene content.

**Fig. 2.**
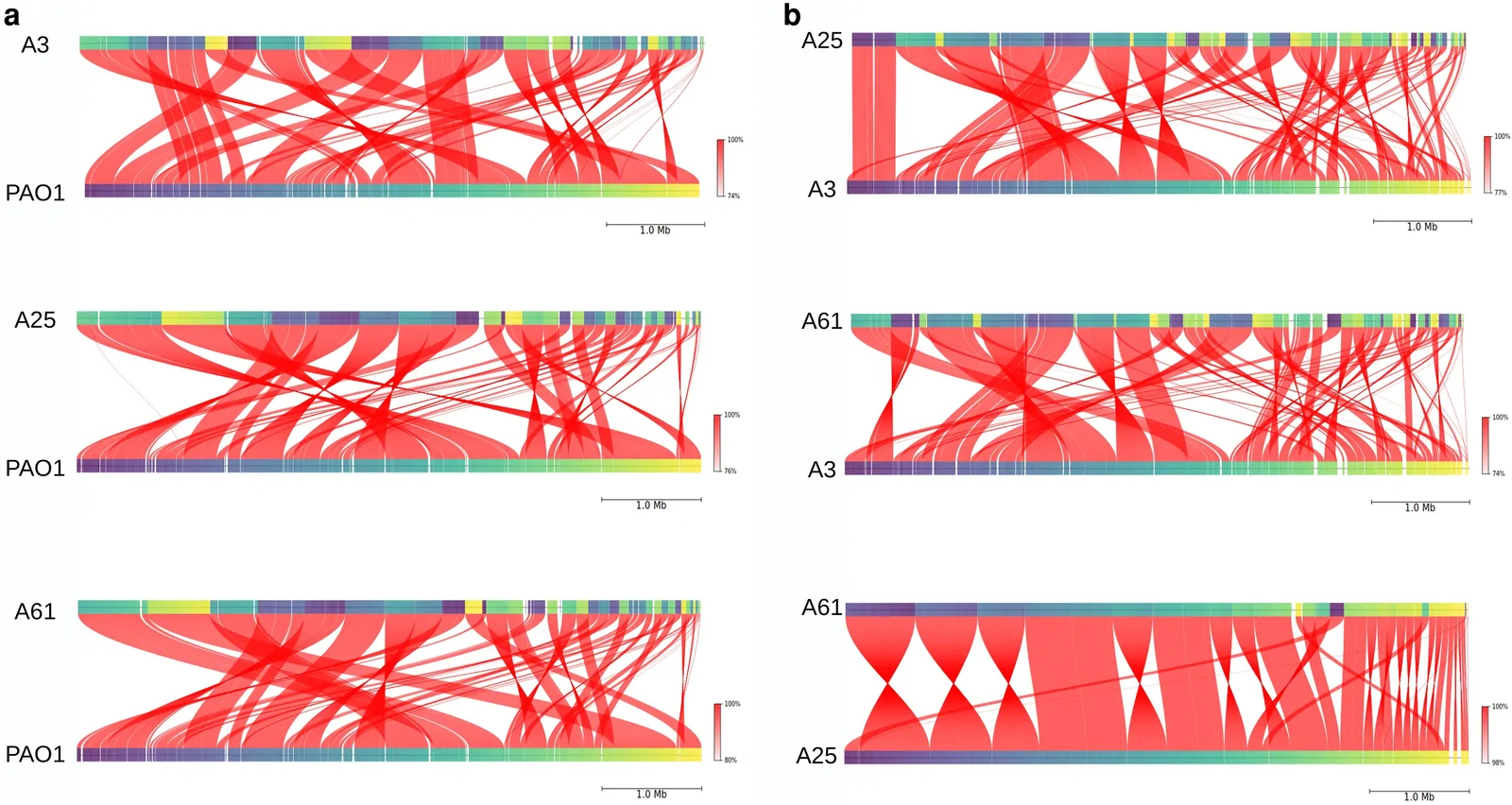
Genome-wide similarity between clinical isolates and the *Pseudomonas aeruginosa* PAO1 reference strain assessed by FastANI. a) Pairwise whole-genome average nucleotide identity (ANI) values calculated using FastANI, showing the degree of genomic similarity between each clinical isolate (A3, A25, and A61) and the *P. aeruginosa* reference genome PAO1. b) FastANI scatterplots illustrating fragment-level alignment identity between each isolate.

We used metagenomics to reconstruct high-quality *P. aeruginosa* genomes from these 3 samples (patients A3, A25, and A61) (Fig. 1a). All 3 patients are male, around 50 yr old, and do not have any underlying condition, such as tuberculosis, bronchiectasis, COPD, allergic bronchopulmonary disease (ABPA), or asthma (Table 1). Patient A3 had hemoptysis, and the size of the aspergilloma varies from 5 to 24 mm, and the duration of symptoms extends from 1.5 to 17 yr (Table 1). Based on 16S and ITS amplicon analysis (Liu et al. 2026), all 3 fungal balls were colonized with about 100% *P. aeruginosa*, while the fungal balls from patients A25 and A61 were colonized with more than 90% *A. fumigatus*, and patient A25 with more than 90% *A. flavus* (Liu et al. 2026). Additional evidence for the identity of *P. aeruginosa* came from taxonomic classification using Kraken2 with the SILVA rRNA release 138 databases, which confirmed that all assemblies belong to the genus *Pseudomonas*, with *P. aeruginosa* as the predominant species. The reconstructed MAGs exhibited genome sizes of 6,368,664 bp (A3), 6,263,885 bp (A25), and 6,243,234 bp (A61), with completeness scores of 99.3% (A3), 98.6% (A25), and 98.9% (A61), and contamination levels below 1.2%, as assessed by CheckM. The total number of contigs varied across samples, with 42 contigs in A3, 36 in A25, and 37 in A61. All genomes exhibited an average GC content of approximately 66.5%, consistent with that of reference *P. aeruginosa* genomes (Supplementary Table 1). The 3 *P*. aeruginosa MAGs recovered from the fungal ball samples (A3, A25, and A61) exhibited a high degree of genomic conservation when compared against the *P. aeruginosa* PAO1 reference genome (Fig. 1b). The BLAST Ring Image Generator (BRIG) analysis revealed concentric rings with >99% nucleotide identity across the majority of the genome, indicating that the core genomic architecture is highly conserved among the 3 isolates and the PAO1 reference strain, consistent with the high pairwise ANI values observed (Fig. 2).

**Table 1.** Clinical metadata for aspergillomas extracted from 3 patients (A3, A25, and A61).

Patients	Gender	Age (yr)	Diameter of fungal balls (mm)	Duration of symptoms (yr)	Haemoptysis	Antifungal therapy	Antibiotic therapy
A3	Male	47	5	17	Yes	None	None
A25	Male	51	24	1.75	No	None	Cephalosporins
A61	Male	51	20	1.5	No	None	Cephalosporin and Quinolones

Together, these results indicate that although the aspergilloma-associated isolates retain the canonical *P. aeruginosa* genomic backbone, subtle genetic differences likely influence niche adaptation, metabolic flexibility, and interactions with both *A. fumigatus* and the human host.

### Virulence factors, antimicrobial resistance determinants, secondary metabolites, and BGCs

Functional annotation using the VFDB (https://www.mgc.ac.cn/VFs/) showed that the 3 MAGs shared approximately 92% of the canonical virulence gene repertoire characteristic of *P. aeruginosa*. These included major categories such as nutritional/metabolic factors, effector delivery systems (T3SS and T6SS), biofilm formation, motility and adherence determinants, and immune-modulatory components (Table 2 and Supplementary Table 1). In total, 304 virulence-associated genes were identified in strains A3 and A25, and 311 in strain A61, of which 280 genes were conserved across all 3 MAGs. Only 1 unique virulence-associated gene was identified per genome (Table 2 and Supplementary Table 1).

**Table 2.** Virulence and secondary metabolites genes identified on MAGs.

Gene classes	Source	Sample A3	Sample A25	Sample A61
Virulence factor	VFDB	304	304	311
Transporter	TCDB	221	213	212
Metal Resistance	BacMet	27	28	29
Drug Target	TTD	19	18	18
Antibiotic Resistance	PATRIC	98	93	94
Antibiotic Resistance	CARD	61	61	61
Antibiotic Resistance	NDARO	5	5	5
Secondary Metabolites	antiSMASH	17	13	15

Antimicrobial resistance profiling was performed using the RGI against the CARD. A total of 61 resistance determinants were identified and shared across the 3 *P. aeruginosa* MAGs (Supplementary Table 1 and Table 2). Regarding clinical history, patient A61 had received cephalosporins and quinolones prior to surgical resection; patient A25 had been treated with cephalosporins only; and patient A3 had not received antibiotic therapy (Table 1). Despite these differences in exposure, genes associated with cephalosporin resistance were detected in all 3 genomes, and fluoroquinolone-associated resistance markers were also identified across the 3 isolates. Although phenotypic antimicrobial susceptibility testing was not performed, the presence of β-lactam and fluoroquinolone resistance determinants in all MAGs suggests that these resistance traits are embedded in the genomic background of the isolates and may reflect intrinsic and/or previously acquired resistance mechanisms rather than solely recent therapeutic selection.

The staramr pipeline further confirmed the presence of 6 conserved AMR genes consistent with multidrug-resistant phenotypes frequently reported in chronic pulmonary infections. Notably, strain A61 harbored an additional ciprofloxacin-resistance gene not present in strains A3 and A25, suggesting that its evolutionary trajectory was likely shaped by local antimicrobial exposure (Supplementary Table 1). The combined presence of multiple efflux systems, β-lactamases, and enzymatic inactivators underscores the high degree of antibiotic resilience in these isolates, reinforcing the notion that *P. aeruginosa* populations in chronic infections acquire extensive resistance traits through sustained selective pressures.

Analysis of BGCs revealed a diverse and functionally rich secondary metabolite repertoire across the 3 MAGs (Table 2 and Supplementary Table 1). In total, 3 clusters were identified, including nonribosomal peptide synthetase (NRPS) systems, phenazine pathways, hydrogen cyanide biosynthetic genes, homoserine lactone-associated quorum-sensing modules, betalactone clusters, and both Type I and Type III polyketide synthases (T1PKS/T3PKS) (Supplementary Table 1). Notably, the A3 genome uniquely contained 2 duplicated PKS clusters, suggesting expanded metabolic potential. Altogether, the secondary metabolite landscape highlights the biochemical versatility of aspergilloma-derived *P. aeruginosa* and supports their capacity for intense interspecies competition, environmental sensing, and survival within the dense fungal matrix.

### Pangenome structure and phylogenomic relationships

To contextualize the reconstructed genomes within the global diversity of *P. aeruginosa*, we performed a comprehensive pangenomic analysis incorporating the 3 aspergilloma-derived MAGs (A3, A25, and A61) together with 648 reference genomes from the BV-BRC (Bacterial and Viral Bioinformatics Resource Center) (https://www.bv-brc.org/) (Fig. 1c and Supplementary Table 2). The combined dataset revealed a pangenome comprising 61,058 gene families, reflecting the high genomic plasticity typical of *P. aeruginosa*. Of these, 2,508 genes constituted the highly conserved core genome (present in ≥99% of isolates), while 1,857 were classified as soft core genes (present in 95 to 99% of genomes), 2,260 genes shell genes (present in 15 to 95% of genomes) and 54,433 cloud genes (present in 0 to 15% of genomes)

Phylogenomic reconstruction revealed notable differences among the 3 MAGs. Samples A25 and A61 clustered closely together, forming a tight clade within a large group of global *P. aeruginosa* strains, suggesting they share a more recent evolutionary history or similar selective pressures. In contrast, sample A3 was positioned more distantly, branching separately from A25 and A61 and exhibiting greater divergence relative to the broader population structure. This phylogenetic separation aligns with the genomic variation observed in accessory and unique gene content among the isolates. Importantly, no clear clustering patterns were observed based on geographical origin (continent) or isolation environment, as the reference strains from diverse regions and ecological niches were broadly intermixed throughout the phylogeny. This lack of biogeographic structure supports previous observations that *P. aeruginosa* exhibits a largely panmictic global population, characterized by extensive horizontal gene transfer and frequent adaptive convergence, rather than phylogenetically distinct geographic lineages. The phylogenomic divergence of A3 and the close clustering of A25 and A61 reflect heterogeneous evolutionary trajectories within the same class of clinical niche.

### Global gene expression profile of *P. aeruginosa* in pulmonary aspergillomas

**Fig. 3.**
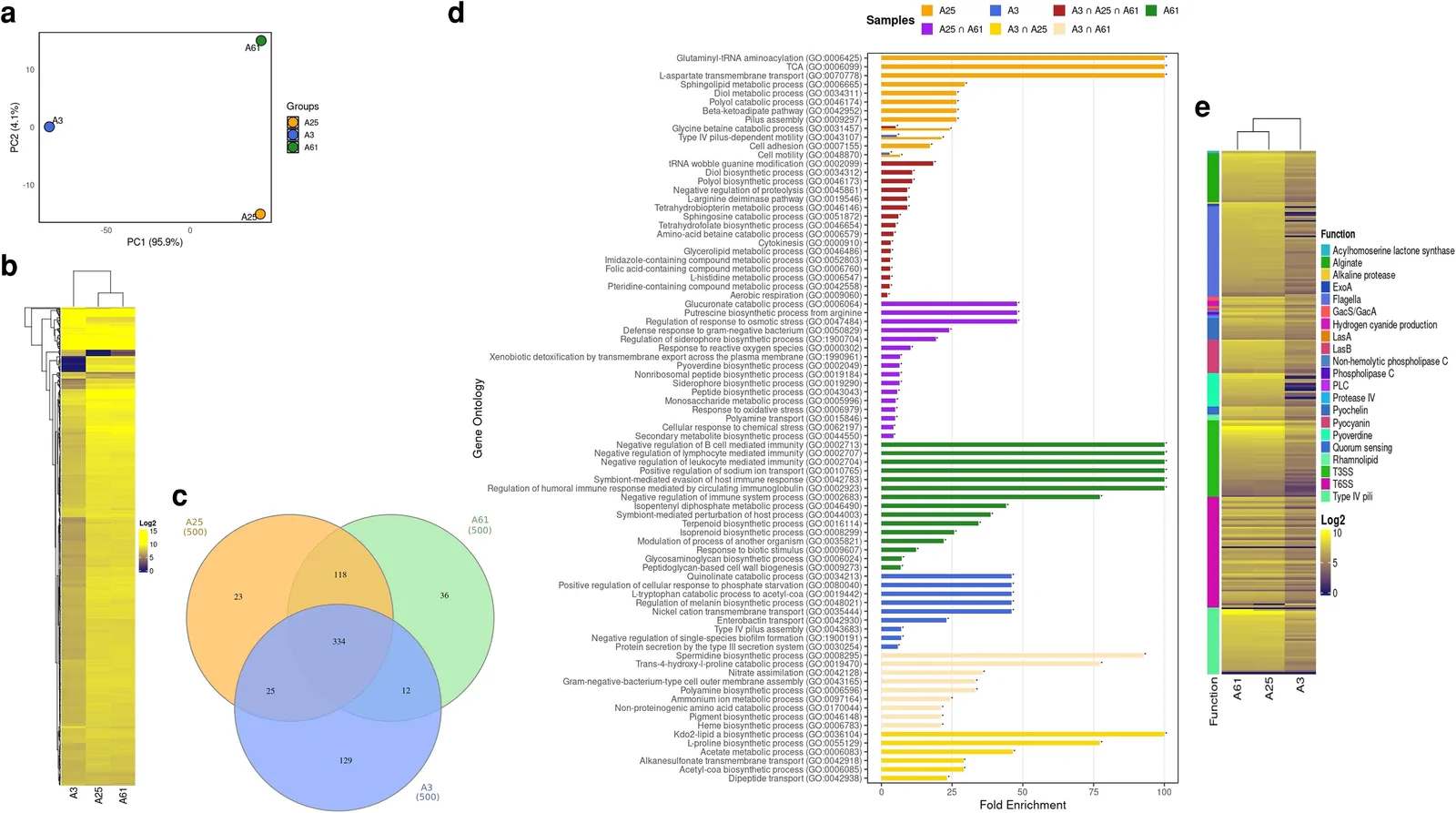
Metatranscriptomic profiling of *Pseudomonas aeruginosa* across 3 aspergilloma samples. a) PCA of normalized TPM values for the 3 *P. aeruginosa*–positive samples (A25, A61, A3). b) Heatmap of the top 500 most variable genes, displayed as log_2_(TPM + 1). c) Venn diagram showing the overlap among the top 500 expressed genes from each sample (A25 = orange, A61 = green, A3 = blue). d) GO enrichment analysis of expressed genes, showing terms with adjusted *P* < 0.05, “∩” symbols indicate terms derived from intersections between samples. e) Heatmap of virulence-related genes, using the same log_2_(TPM + 1) transformation and color scale as in panel b.

Metatranscriptomic profiling of 3 *P. aeruginosa* isolates (A3, A25, A61) revealed marked heterogeneity in global gene expression despite their presence in structurally similar aspergillomas (Fig. 3). After TPM normalization, PCA demonstrated clear separation of the 3 isolates, indicating distinct transcriptional states. A25 and A61 were highly correlated (Spearman ρ = 0.99) yet remained separable, whereas A3 showed lower similarity to both (ρ ≈ 0.80), consistent with a more divergent expression profile.

Analysis of the top 500 expressed genes reinforced this divergence. A3 lacked expression of multiple genes strongly expressed in A25 and A61, including the bacteriocin pyocin S5, suggesting reduced engagement in interbacterial competition. Conversely, A25 uniquely lacked expression of specific adhesion-related genes, including *fimT*. A shared core of 334 genes was expressed across all isolates, representing conserved metabolic and structural functions, but A3 displayed the largest fraction of unique transcripts (∼25%), indicating a more specialized or constrained physiological program.

Functional enrichment analysis further distinguished the isolates. A3 was enriched for stress adaptation, iron acquisition, motility, and biofilm-associated pathways, consistent with niche adaptation under environmental constraints. A25 showed enrichment in central carbon metabolism (TCA cycle), lipid-related pathways, and adhesion functions, suggesting a metabolically active state. A61 exhibited enrichment in immune-modulatory and stress-response pathways, including terpenoid biosynthesis and responses to external stimuli, indicating intensified interaction with host-derived signals. Core pathways such as aerobic respiration, L-arginine metabolism, and folate metabolism were conserved across isolates.

Virulence-associated operons displayed pronounced differences. A25 and A61 strongly expressed siderophore biosynthesis genes (*pvd*, *pch*), type IV pili components, pyocins, phenazine biosynthesis genes, and type III secretion system (T3SS) regulators and effectors. In contrast, A3 showed reduced or absent expression across many of these systems. T6SS components were particularly enriched in A61, suggesting enhanced interbacterial or host interaction. Overall, A25 and A61 exhibited a broad and active virulence repertoire, whereas A3 displayed an attenuated profile, consistent with transcriptional plasticity shaped by microenvironmental conditions.

### Host transcriptional response

**Fig. 4.**
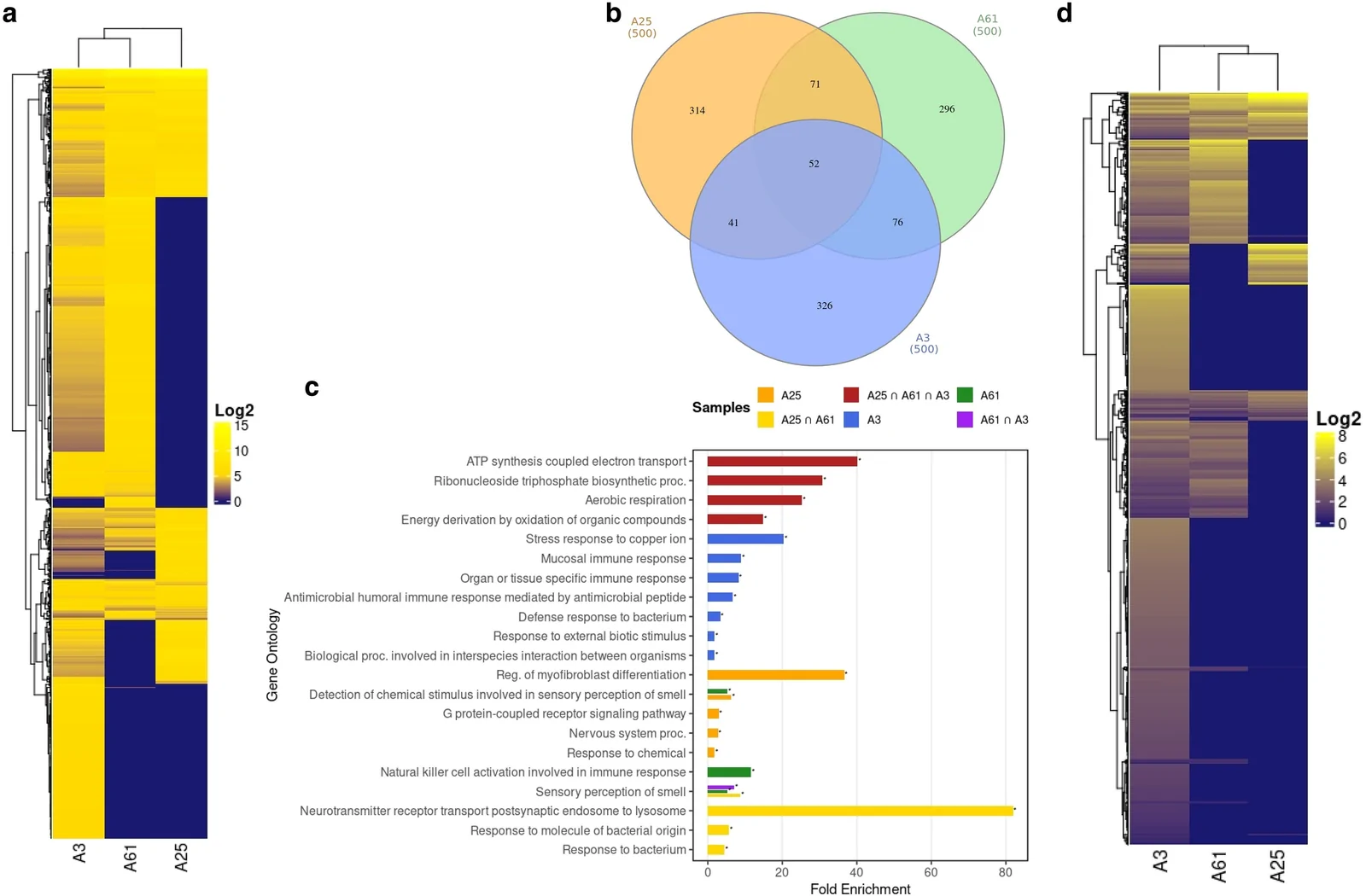
Host transcriptomic profiling across aspergilloma samples. a) Heatmap of the top 500 most variable human genes, displayed as log_2_(TPM + 1). b) Venn diagram showing the overlap of expressed genes among the 3 samples. c) GO enrichment analysis of expressed genes, showing terms with adjusted *P* < 0.05, “∩” symbols indicate terms derived from intersections between samples. d) Heatmap of 1,200 immune-related genes, plotted as log_2_(TPM + 1) using the same color scale as in panel a.

Human transcriptomes also exhibited strong inter-individual variability (Fig. 4). A25 and A61 showed the highest similarity (ρ = 0.88), whereas A3 was more distinct. Only 52 genes were shared across all samples, and more than 60% of expressed genes were patient-specific, highlighting substantial heterogeneity in host responses. All samples shared activation of core metabolic pathways, but enrichment patterns differed. A3 was enriched for mucosal immune activation and bacterial defense pathways. A25 showed signatures associated with tissue remodeling and moderate immune activation. A61 exhibited enrichment for immune cell death and inflammatory responses, consistent with a highly reactive microenvironment.

Analysis of 1,045 curated immune-related genes confirmed minimal overlap between samples (pairwise ρ values near zero). A3 displayed broad immune activation, A25 showed limited immune gene expression (∼17% of curated genes), and A61 demonstrated substantial activation (∼40%), aligning with the higher bacterial virulence activity observed in this sample. These findings indicate that host transcriptional states closely mirror the physiological and virulence profiles of the resident *P. aeruginosa* populations.

### Metatranscriptomic activity of *A. fumigatus*

**Fig. 5.**
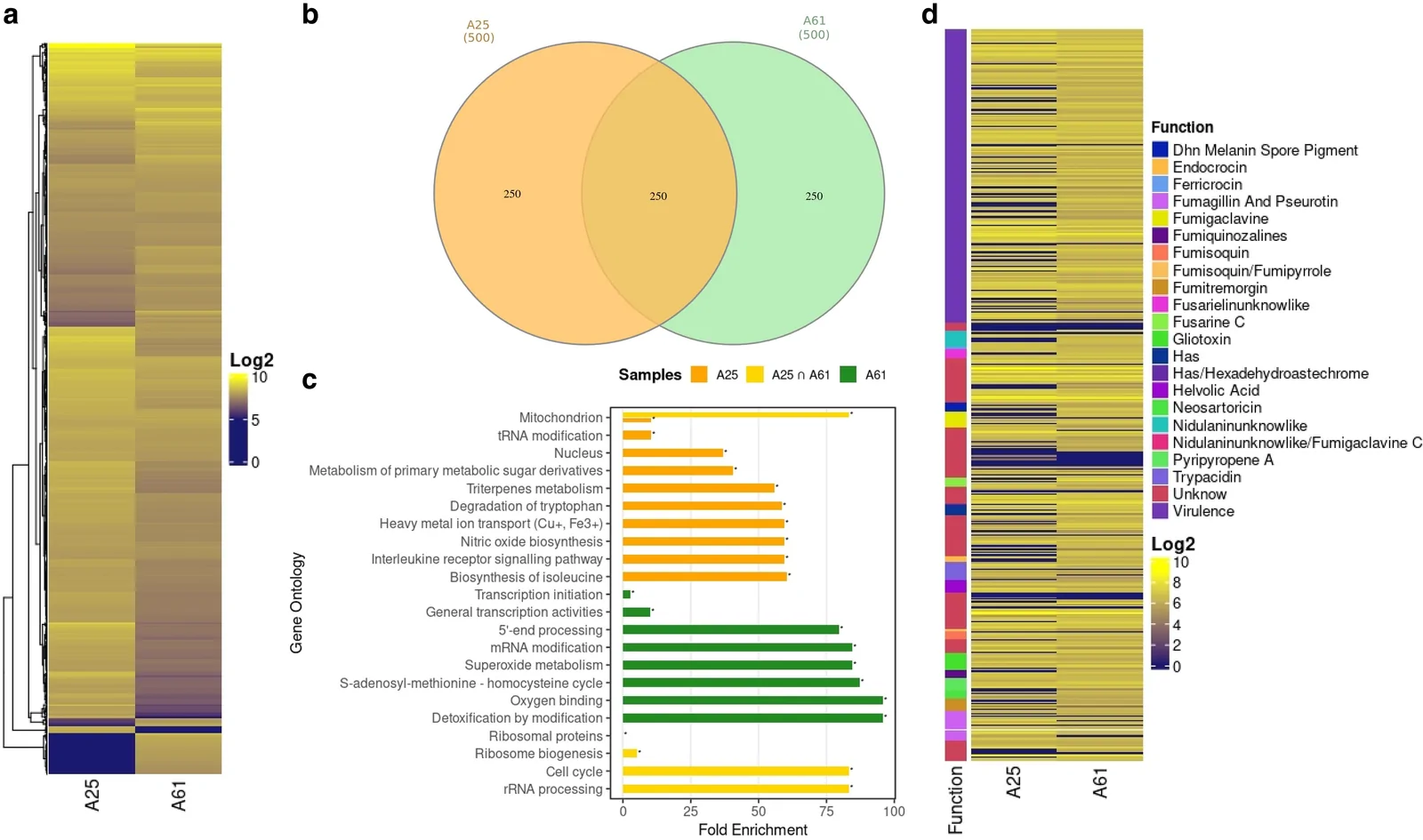
Transcriptomic profiling of *Aspergillus fumigatus* within aspergillomas. a) Heatmap of the top 500 most highly expressed fungal genes, shown as log_2_(TPM + 1). b) Venn diagram illustrating the overlap of expressed genes between isolates A25 and A61. c) GO enrichment analysis of expressed genes, showing terms with adjusted *P* < 0.05, “∩” symbols indicate terms derived from intersections between samples. d) Heatmap of virulence-associated genes plotted as log_2_(TPM + 1) using the same color scale as in panel a.

Fungal genome reconstruction was not possible due to extremely low fungal read abundance (<0.05% of total reads), as confirmed by Kraken2 profiling and absence of BUSCO completeness, indicating insufficient genomic coverage rather than technical failure. Fungal transcriptomic analysis was therefore restricted to A25 and A61. Although global profiles were broadly similar, hierarchical clustering and gene overlap analysis revealed isolate-specific expression patterns (Fig. 5a). Approximately half of the expressed genes were shared, with the remainder uniquely expressed in each sample (Fig. 5b)

Functional enrichment analysis showed that A25 favored primary metabolic pathways, including terpenoid biosynthesis and metal transport, suggesting metabolic activity and host interactions (Fig. 5c). In contrast, A61 was enriched for oxidative stress responses and detoxification pathways, consistent with a more inflammatory microenvironment (Fig. 5c). Core processes such as ribosomal function and mitochondrial metabolism were conserved (Fig. 5c). Virulence-associated genes were more highly expressed in A61 than in A25, paralleling the stronger bacterial virulence program and heightened host immune activation in this sample (Fig. 5d). Together, these data indicate coordinated modulation of fungal, bacterial, and host transcriptional programs within each aspergilloma, with A61 representing the most transcriptionally interactive and immunologically active microenvironment.

### Metabolomic profiling of Aspergilloma samples reveals distinct chemical environments and host–microbe interactions

**Fig. 6.**
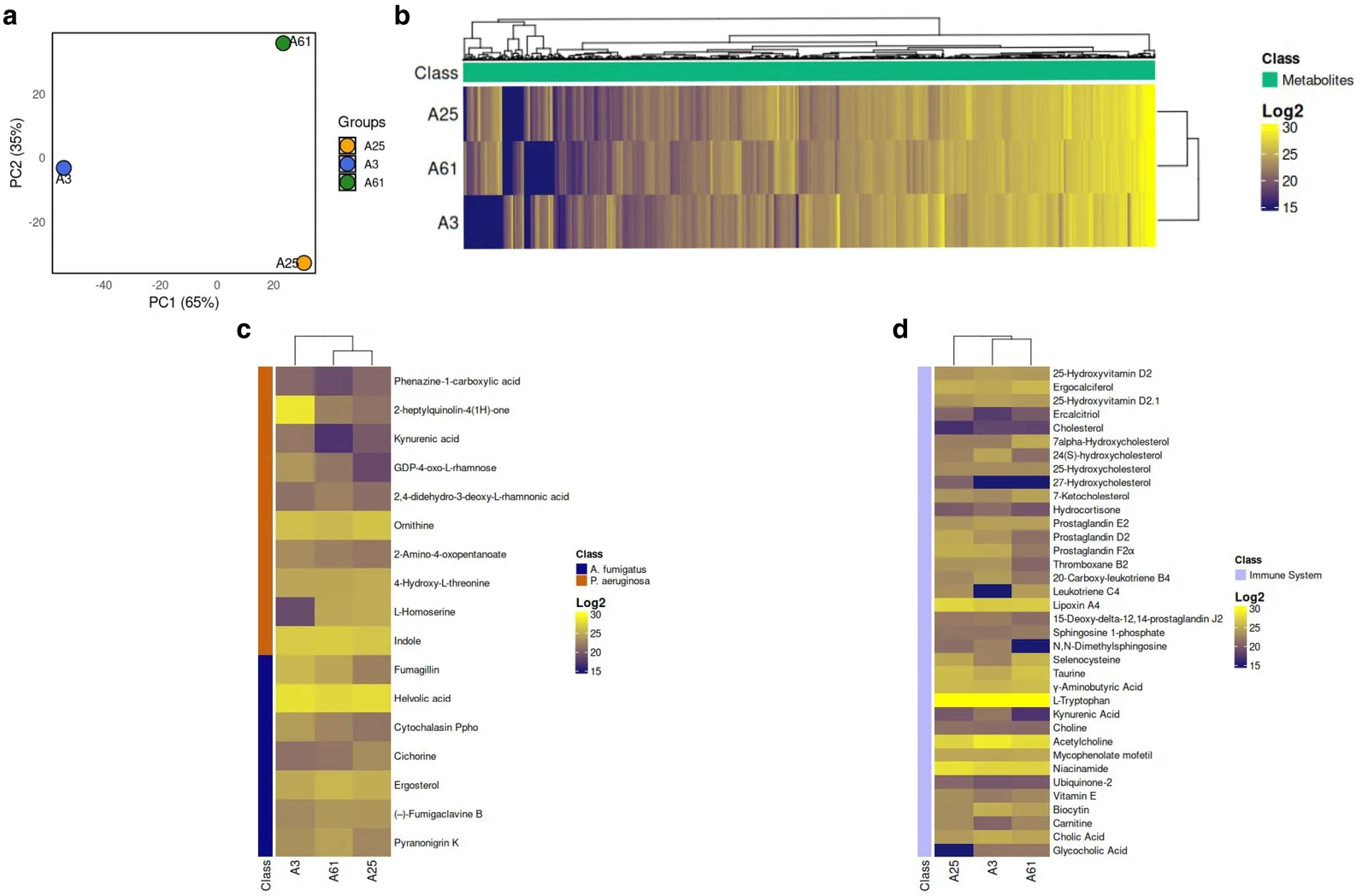
Untargeted metabolomics profiling of fungal ball-associated aspergilloma samples. a) PCA showing the global distribution of metabolite profiles across samples, illustrating clear separation driven by biological variation among conditions. b) Heatmap of all curated metabolites (log_2_-transformed peak intensities), following database-based annotation and manual curation. c) Focused heatmap highlighting metabolites associated with *Aspergillus fumigatus* and *Pseudomonas aeruginosa* (log_2_-transformed peak intensities). d) Heatmap of metabolites linked to host immune processes (log_2_-transformed peak intensities), emphasizing immune-associated metabolic responses within the aspergilloma microenvironment.

Untargeted metabolomic profiling identified a total of 3,418 compounds, which were subsequently filtered using organism-specific databases for *A. fumigatus* (Aspergillus Metabolome Database) (https://www.aspergillusmetabolome.org/), *P. aeruginosa* (Pseudomonas Metabolome Database) (http://pseudomonas.umaryland.edu/), and Human Metabolome Database (https://www.hmdb.ca/) (Supplementary Table 4). All candidate metabolites were then manually curated to ensure high-confidence biological assignments, excluding non-biological artifacts and validating each metabolite against known fungal, bacterial, and host metabolic pathways. This integrative annotation revealed distinct metabolomic signatures across the 3 aspergilloma samples. Spearman correlations showed moderate similarity between A3–A25 (0.74) and A3–A61 (0.73), whereas A25–A61 displayed a higher degree of overlap (0.86), indicating a partially shared chemical environment between these 2 samples (Fig. 6a, b, Supplementary Table 4 and Supplementary Fig. 1e). These differences likely reflect variation in the underlying microbial community composition, metabolic activity, and host–microbe interactions within each fungal ball.

Across the curated metabolome, several high-confidence metabolites emerged as dominant biochemical signatures of the bacterial, fungal, and host compartments. *P. aeruginosa* displayed a metabolomic profile centered on virulence regulation, redox activity, and adaptive nitrogen metabolism (Fig. 6b and Supplementary Table 4). The strongest markers included phenazine-1-carboxylic acid, a key precursor in the phenazine network driving oxidative stress, biofilm maturation, and antifungal antagonism; 2-heptylquinolin-4(1H)-one (HHQ), a core PQS signaling molecule; and kynurenic acid, reflecting bacterial tryptophan catabolism (Fig. 6b and Supplementary Table 3). Additional metabolites such as ornithine, 4-hydroxy-L-threonine, L-homoserine, 2-amino-4-oxopentanoate, and rhamnose-pathway intermediates (GDP-4-oxo-L-rhamnose, 2,4-didehydro-3-deoxy-L-rhamnonic acid) indicate active remodeling of nitrogen flux, amino acid turnover, and exopolysaccharide biosynthesis (Fig. 6b and Supplementary Table 3). The detection of indole further suggests interkingdom signaling with potential effects on fungal physiology and host responses.

We identified in the *A. fumigatus* metabolome canonical virulence-associated secondary metabolites (Fig. 6c and Supplementary Table 4). Fumagillin and helvolic acid potent inhibitors of neutrophil and epithelial function were among the most abundant fungal markers. Additional toxins and bioactive molecules such as cytochalasin P, cichorine, fumigaclavine B, and pyranonigrin K point to strong activation of multiple secondary-metabolite biosynthetic clusters, consistent with the transcriptomic enrichment of detoxification and oxidative-stress pathways (Fig. 6c and Supplementary Table 4). Structural sterols, particularly ergosterol, highlight active membrane remodeling characteristic of fungal adaptation in hypoxic airway niches (Fig. 6c and Supplementary Table 4).

Host-derived metabolites reflected intense activation of inflammatory, immunomodulatory, and lipid-signaling pathways (Fig. 6d and Supplementary Table 4). Eicosanoids emerged as the dominant immune mediators, including prostaglandin E2, prostaglandin D2, prostaglandin F2α, thromboxane B2, leukotriene C4, 20-carboxy-LTB4, and the pro-resolution mediator lipoxin A4, mapping directly onto transcriptomic signatures of neutrophil recruitment, vascular activation, and inflammation resolution (Fig. 6d and Supplementary Table 4). Oxysterols involved in innate immune regulation, 25-hydroxycholesterol, 27-hydroxycholesterol, 7-ketocholesterol, and 24(S)-hydroxycholesterol were represented, alongside hydrocortisone, indicating glucocorticoid-linked modulation of inflammation. Immune-relevant amino acid derivatives, including L-tryptophan, kynurenic acid, taurine, selenocysteine, and γ-aminobutyric acid, further reflect coordinated metabolic reprogramming within activated immune cells. Lipid and membrane-associated molecules such as sphingosine-1-phosphate, N,N-dimethylsphingosine, choline, acetylcholine, and vitamin-derived antioxidants (vitamin E, niacinamide, ubiquinone-2) highlight additional layers of immune signaling, oxidative defense, and epithelial homeostasis. Bile acids and conjugates, including cholic acid and glycocholic acid, further implicate host metabolic pathways linked to mucosal immunity and inflammatory modulation.

## Discussion

Chronic pulmonary aspergillomas are structured, hypoxic, and nutritionally restricted lesions in which fungi, bacteria, and host cells coexist for prolonged periods. Although *A. fumigatus* is the principal etiological agent of fungal balls, bacterial co-colonization, particularly by *P. aeruginosa* is common and clinically relevant. Cohort studies have associated the presence of P. aeruginosa with more severe disease and poorer outcomes in CPA and cavitary lung conditions (Reece et al. 2018; Keown et al. 2020). Experimental systems have consistently demonstrated that *P. aeruginosa* and *A. fumigatus* engage in direct antagonism. Phenazines impair fungal mitochondrial respiration and promote oxidative stress (Briard et al. 2015), PQS-family quinolones alter fungal physiology (Reen et al. 2016), and pyoverdine-mediated iron sequestration restricts fungal growth under iron limitation (Sass et al. 2018). Conversely, *A. fumigatus* produces secondary metabolites, including gliotoxin and helvolic acid, that modulate bacterial proliferation and host immunity (Sass et al. 2018; Steenwyk et al. 2020). Importantly, murine coinfection models indicate that these interactions are not restricted to in vitro systems: co-infected animals show altered fungal burden and survival compared with monoinfection, demonstrating that interkingdom competition reshapes disease trajectories in vivo (Briard et al. 2019).

Within this conceptual framework, our genome-resolved analysis indicates that *P. aeruginosa* in aspergillomas represents a stable, adapted population rather than incidental contamination. Chronic pulmonary infections, most extensively characterized in CF, demonstrate that *P. aeruginosa* undergoes progressive within-host diversification, accumulation of resistance determinants, and remodeling of regulatory and virulence circuits during long-term persistence (Lozano et al. 2018; Abram et al. 2022). The combination of a highly conserved core genome and variable accessory content observed in our isolates aligns with the species’ open pangenome architecture and its well-documented capacity for rapid niche adaptation (Corredor et al. 2024). Moreover, the coexistence of phylogenetically distinct lineages within the same pulmonary environment has been described in CF lungs (Markussen et al. 2014; Williams et al. 2015), reinforcing that localized selective pressures drive microevolutionary divergence even within structurally confined lesions.

Antimicrobial exposure represents one of the most potent selective forces shaping this evolutionary trajectory. *P. aeruginosa* displays intrinsic resistance to many β-lactams, including first- and second-generation cephalosporins and several third-generation agents, primarily due to constitutive or inducible expression of the chromosomal AmpC β-lactamase, reduced outer membrane permeability, and multidrug efflux systems such as MexAB-OprM. In contrast, resistance to fluoroquinolones is typically acquired and arises through stepwise mutations in the quinolone-resistance-determining regions of *gyrA/gyrB* and *parC/parE*, frequently accompanied by efflux pump overexpression or reduced porin expression (Strateva and Yordanov 2009). Resistance rates reported globally further contextualize these findings. A recent systematic review spanning Asia and Africa (2018 to 2023) documented ciprofloxacin resistance rates ranging from approximately 30% to 70%, depending on region and clinical setting (Salleh et al. 2025). Similarly, in our dataset, the identification of multiple β-lactam resistance determinants across all MAGs, together with fluoroquinolone-associated resistance elements in at least 1 isolate, is consistent with this established adaptive repertoire.

At the functional level, the integration of transcriptomic and metabolomic data supports deployment of established pathogenic programs within aspergillomas. Upregulation of phenazine and quinolone biosynthesis aligns with prior in vivo transcriptomic studies demonstrating enrichment of these pathways in sputum and animal infection models relative to laboratory growth conditions (Cornforth et al. 2018). Phenazine production is a recognized adaptation to oxygen-limited environments, facilitating redox balancing and biofilm resilience (Harrington et al. 2022). Given the demonstrated antifungal activity of phenazines (Briard et al. 2015), their in situ expression likely contributes to spatial restriction of fungal growth within the cavity.

Iron competition represents another mechanistically grounded axis. Both bacterial siderophores (pyoverdine, pyochelin) and fungal hydroxamate siderophores are strongly induced during mammalian infection (Reece et al. 2018; Liu et al. 2021). Nutritional immunity in the lung further limits iron availability, creating a competitive landscape in which siderophore systems become essential for persistence. The concordant activation of iron-acquisition genes across kingdoms is therefore consistent with established models of bacterial–fungal competition under host-imposed iron restriction rather than speculative cross-talk. Metabolic remodeling toward nitrogen scavenging and amino acid catabolism similarly reflects well-characterized adaptations of *P. aeruginosa* in nutrient-depleted chronic lung environments (Turner et al. 2014). In CF sputum, reliance on host-derived amino acids and peptides supports long-term persistence and biofilm maturation. The patterns observed here are congruent with that paradigm, suggesting that aspergillomas impose comparable metabolic constraints.

The fungal response also conforms to experimentally validated infection biology. During pulmonary infection, *A. fumigatus* upregulates oxidative-stress defenses, siderophore biosynthesis, zinc transporters, and nitrogen assimilation pathways (Liu et al. 2021). Reactive oxygen species generated by neutrophils and inflamed epithelium create sustained oxidative pressure (Shankar et al. 2018). Induction of catalases, superoxide dismutases, and glutathione-dependent systems therefore reflects canonical fungal adaptation to host immunity. Activation of secondary metabolite clusters producing fumagillin, helvolic acid and related compounds aligns with their documented roles in immunomodulation, angiogenesis inhibition and microbial antagonism (Steenwyk et al. 2020; Arastehfar et al. 2021). Rather than representing passive colonization, these transcriptional programs indicate metabolically active fungal populations engaged in defense and resource competition.

Host responses further mirror known immunopathological pathways in chronic pulmonary fungal infection. Enrichment of eicosanoid synthesis genes (eg PTGS2, ALOX5) and detection of prostaglandins and leukotrienes correspond to established inflammatory circuits driving neutrophil recruitment and vascular permeability in aspergillosis (Kale et al. 2017). Recognition of fungal β-glucans via dectin-1 and Toll-like receptors activates NF-κB and Jak–STAT signaling cascades (Shankar et al. 2018), promoting Th1/Th17 polarization. Concurrent activation of the tryptophan–kynurenine pathway, mediated by IDO1, has been shown to modulate T-cell responses and promote immune tolerance during fungal infection (Montagnoli et al. 2006). The coexistence of pro-inflammatory lipid mediators and immunoregulatory kynurenines is therefore consistent with a chronic inflammatory state characterized by simultaneous activation and counter-regulation.

Taken together, these findings reinforce a model in which aspergillomas function as metabolically active, competitive ecosystems rather than inert fungal aggregates. Many of the transcriptional and metabolic features observed here, phenazine production, siderophore competition, oxidative-stress adaptation, and eicosanoid-driven inflammation have been independently validated in infection models. Their convergence within resected human tissue provides in vivo confirmation that canonical bacterial–fungal antagonistic mechanisms operate within chronic pulmonary cavities. By integrating genome-resolved metagenomics with matched transcriptomic and metabolomic profiling, this study situates aspergillomas within a broader framework of chronic polymicrobial biofilm infections, where evolutionary plasticity, metabolic competition, and host immunoregulation collectively sustain long-term persistence.

## Conclusion

This study provides the first integrated multi-omics characterization of *P. aeruginosa* adaptation within the ecological niche of the aspergilloma. By combining high-quality MAGs with matched metatranscriptomic, metabolomic, and host transcriptional data, we demonstrate that *P. aeruginosa* acts as an active and genomically plastic member of a tri-kingdom ecosystem rather than a passive colonizer. Bacterial persistence is supported by a conserved core genome alongside accessory elements encoding virulence, antimicrobial resistance, and specialized metabolic pathways shaped by chronic pulmonary selective pressures. Phylogenomic reconstruction revealed distinct evolutionary trajectories among co-colonizing strains, while transcriptional profiling uncovered heterogeneous physiological states, indicating substantial regulatory plasticity in vivo. The concurrent detection of phenazines and quorum-sensing metabolites, together with fungal oxidative stress responses and host immune activation signatures, confirms active interkingdom metabolic and chemical interactions within aspergillomas.

These findings redefine aspergillomas as dynamic polymicrobial consortia characterized by coordinated microbial competition and host immune modulation, underscoring the need to study chronic pulmonary infections as integrated ecological systems. A limitation of the present study is the lack of spatial resolution between central and peripheral regions of the fungal ball, future application of imaging mass spectrometry and spatial transcriptomics will be essential to determine whether transcriptional programs and metabolite production are spatially structured. Despite cohort size and biomass constraints, this work establishes a multi-omics framework for dissecting polymicrobial biofilm infections in human tissues and provides a foundation for targeting interactive microbial-host networks in chronic infectious diseases.

## Supplementary Material

jkag063_Supplementary_Data

## Data Availability

All sequencing data, including raw reads, assembled genomes, annotations, and metadata, have been deposited in the NCBI Sequence Read Archive under the BioProject accession number: PRJNA1344265 (amplicon-seq data) and PRJNA1368994 (metagenome data), and the metabolomics data, https://doi.org/10.5281/zenodo.17402277 (metabolomics). Supplemental material available at G3 online.
