# Correction to: Pseudomonas aeruginosa adaptation and persistence in the aspergilloma microbiome revealed by integrated multi-omics

**DOI:** 10.1093/g3journal/jkag236

**Published:** 2026-09-04

This is a **correction** to:

Matheus Mertz Ribeiro, Chan Liu, Jin-Fu Xu, Shuo Liang, Gustavo H Goldman, *Pseudomonas aeruginosa* adaptation and persistence in the aspergilloma microbiome revealed by integrated multi-omics, *G3 Genes|Genomes|Genetics*, Volume 16, Issue 5, May 2026, jkag063, https://doi.org/10.1093/g3journal/jkag063

In the originally published version of this manuscript, a couple of author designation and contributions were omitted:

Matheus Mertz Ribeiro and Chan Liu, contributed equally to this work and are co-first authors. In addition, Shuo Liang is a corresponding author alongside Jin-Fu Xu and Gustavo H Goldman.

This error has been corrected.

