# Supplementary material for: Pseudomonas aeruginosa adaptation and persistence in the aspergilloma microbiome revealed by integrated multi-omics
Source: G3 (Bethesda). 2026 Mar 17;16(5):jkag063. doi: 10.1093/g3journal/jkag063 (PMC13148400; doi:10.1093/g3journal/jkag063)
Supplement: jkag063_Supplementary_Data [file jkag063_supplementary_data.zip › Supplemental_Figure_1_G3-2026-406553.pdf]

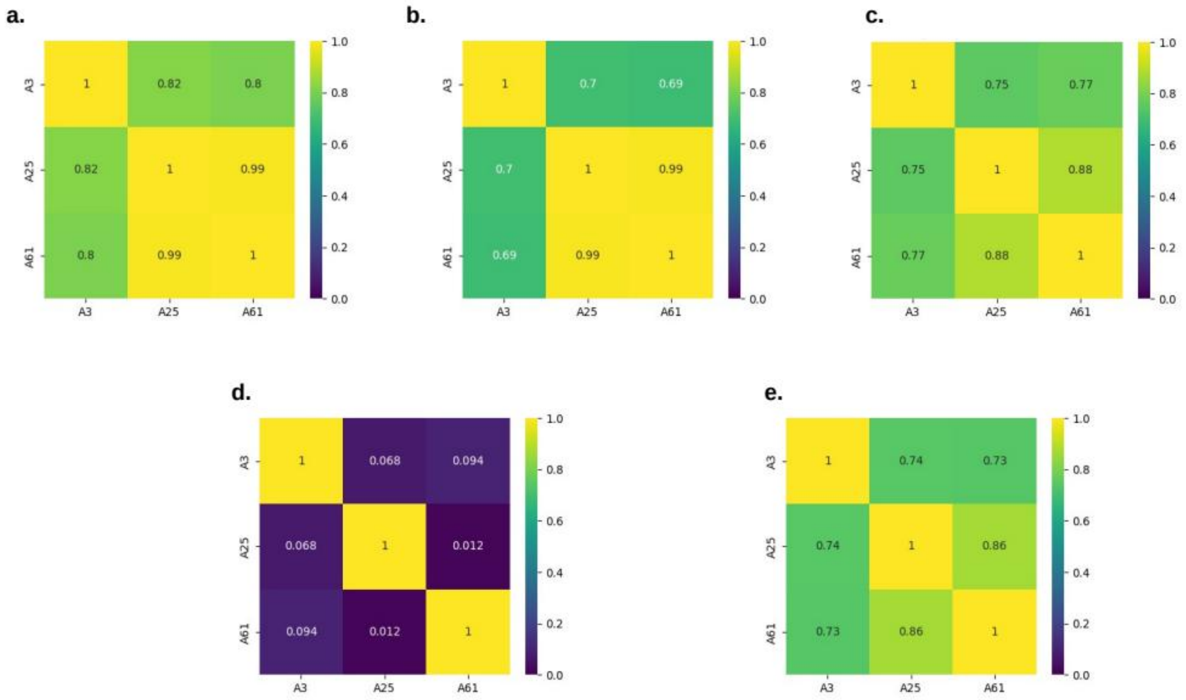

Supplementary Figure 1. Spearman correlation analyses across multi-omic datasets. (a) Pairwise Spearman correlations of global *P. aeruginosa* transcriptomes. (b) Correlation matrix of *P. aeruginosa* virulence gene expression profiles. (c) Spearman correlations of host transcriptomes based on the top 500 most abundant human transcripts. (d) Correlation analysis of host immune-related gene expression (n = 1,045 curated genes). (e) Pairwise Spearman correlations of untargeted metabolomic profiles across aspergilloma samples.
